# Inhibition of ERK 1/2 kinases prevents tendon matrix breakdown

**DOI:** 10.1038/s41598-021-85331-1

**Published:** 2021-03-25

**Authors:** Ulrich Blache, Stefania L. Wunderli, Amro A. Hussien, Tino Stauber, Gabriel Flückiger, Maja Bollhalder, Barbara Niederöst, Sandro F. Fucentese, Jess G. Snedeker

**Affiliations:** 1 Department of Orthopedics, Balgrist University Hospital, University of Zurich, Zurich, Switzerland; 2 Institute for Biomechanics, ETH Zurich, Zurich, Switzerland

**Keywords:** Cell signalling, Proteolysis, Kinases, Proteases, Biochemistry, Cell biology

## Abstract

Tendon extracellular matrix (ECM) mechanical unloading results in tissue degradation and breakdown, with niche-dependent cellular stress directing proteolytic degradation of tendon. Here, we show that the extracellular-signal regulated kinase (ERK) pathway is central in tendon degradation of load-deprived tissue explants. We show that ERK 1/2 are highly phosphorylated in mechanically unloaded tendon fascicles in a vascular niche-dependent manner. Pharmacological inhibition of ERK 1/2 abolishes the induction of ECM catabolic gene expression (MMPs) and fully prevents loss of mechanical properties. Moreover, ERK 1/2 inhibition in unloaded tendon fascicles suppresses features of pathological tissue remodeling such as collagen type 3 matrix switch and the induction of the pro-fibrotic cytokine interleukin 11. This work demonstrates ERK signaling as a central checkpoint to trigger tendon matrix degradation and remodeling using load-deprived tissue explants.

## Introduction

Tendon is a musculoskeletal tissue that transmits muscle force to bone. To accomplish its biomechanical function, tendon tissues adopt a specialized extracellular matrix (ECM) structure^1^. The load-bearing tendon compartment consists of highly aligned collagen-rich fascicles that are interspersed with tendon stromal cells. Tendon is a mechanosensitive tissue whereby physiological mechanical loading is vital for maintaining tendon architecture and homeostasis^2^. Mechanical unloading of the tissue, for instance following tendon rupture or more localized micro trauma, leads to proteolytic breakdown of the tissue with severe deterioration of both structural and mechanical properties^3–5^. These mechanisms are implicated in failed healing response and longer-term tendinopathies, with associated individual pain and socioeconomic burden that afflicts our increasingly aging society^6,7^. At present, the underlying molecular mechanisms of tendinopathies are unknown and present a major roadblock to achieving targeted and effective therapies^8–10^.

In the search for mechanisms that are involved in tendon degradation, tendon explants offer a well-suited tissue model^11,12^. Tendon explants retain features of native tissue including the ECM architecture/structure, allow for highly controlled experimental conditions, and enable direct functional (mechanical) testing. In this sense, tendon explants overcome important limitations of traditional 2D cell culture systems, engineered 3D tissues and in vivo animal models^13^. Ex vivo, tendon explants lose their mechanical properties under load-deprived conditions due to proteolytic matrix degradation involving different proteases, such as matrix metalloproteases (MMP)^12,14–18^. We have previously used murine tendon fascicles to show that vascular like niches drive a cellular stress response and proteolytic tendon degradation^12^. However, the molecular pathways that govern ECM degradation and the subsequent loss of tendon biomechanical function remain unknown. In this work, we identify and probe the extracellular-signal regulated kinases ERK 1/2 as a central checkpoint that regulates proteolytic tendon matrix breakdown.

## Results

**Figure 1:**
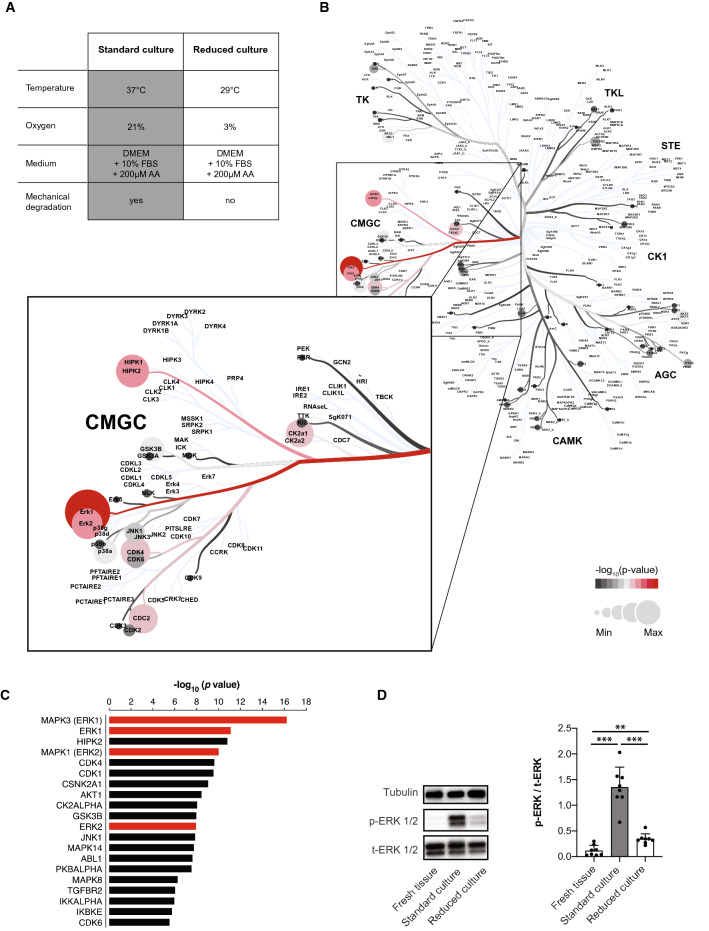
Niche-dependent ERK 1/2 activity and phosphorylation in load-deprived tendon explant cultures. (**A**) Overview of experimental conditions and starting phenotypes. DMEM = Dulbecco's modified Eagle's medium, FBS = fetal bovine serum, AA = ascorbic acid. (**B**) Upstream kinase enrichment prediction based on the transcriptome data set E-MTAB-7832 (ArrayExpress), which analyses differentially expressed genes in tendon tissues cultured ex vivo in standard and reduced niches. Kinome tree dendrogram mapping of all the predicted protein kinases generated by Coral^42^. The circle color and size reflect the enrichment significance with red color encoding higher significance. (**C**) Bar plots show the 20 most significantly enriched kinases. Red bars represent the ERK 1 and 2 kinases. GraphPad Prism (version 8.4.3) was used to generate the figure. (**D**) Western blot validation of ERK 1/2 phosphorylation (ERK 1: Thr202/Tyr204; ERK 2: Thr185/Tyr187) in freshly isolated or cultured tendon fascicles (12 days). Full Western blot images are provided in the supplementary material (Supplementary Fig. 3). Quantification of band pixel intensities of phosphorylated ERK (p-ERK 1/2) compared to the total ERK (t-ERK). GraphPad Prism (version 8.4.3) was used to perform statistical analyses and generate the figure. Bars show mean values + SD, with individual replicate values as data points, N = 8. Repeated measure ANOVA with Tukey's multiple comparisons test: **p < 0.01, ***p < 0.001.

Standard ex vivo tissue culture of tendon explants is widely documented to provoke tissue deterioration. In contrast to the nutrient hyper-availability that characterizes standard tissue culture conditions, we employed more physiological ex vivo culture conditions comprising standard culture medium but at 3% oxygen and 29 °C—conditions we have identified to maintain tendon fascicle viability while preserving native biomechanical properties^12^ (Fig. 1A). Here, we extend these findings by exploring the potential molecular mechanisms that underpin the niche-dependent tendon degeneration in this model system.

We started by re-examining our published transcriptome dataset of tendon fascicles (E-MTAB-7832), in which we have identified ~ 3000 genes to be differentially expressed between standard and reduced niche conditions. First, we applied the Expression2Kinase (E2K) pipeline to computationally infer key regulatory kinases upstream of the differentially expressed genes (DEG). E2K algorithm predicted ~ 100 upstream protein kinases that are likely responsible for regulating DEG. Mapping the identified kinases into Kinome phylogenetic tree showed that the majority of these kinase hits clustered around the CMGC kinases, which is a large kinase group including the mitogen-activated protein kinase (MAPK) family (Fig. 1B). The most significantly enriched kinases are depicted in red color and the MAPK members ERK 1/2 were among the 5 most significantly enriched kinases (Fig. 1B,C, Supplementary table 1). Taking this into consideration, we reasoned that ERK 1/2 may be centrally involved in the niche-dependent degradation of tendon fascicles. Next, we experimentally validated ERK 1/2 predictions using Western Blot analysis and indeed found that the ERK kinases 1/2 are phosphorylated under standard culture conditions but not under reduced culture conditions nor in native, uncultured fascicles (fresh tissue) (Fig. 1D).

**Figure 2:**
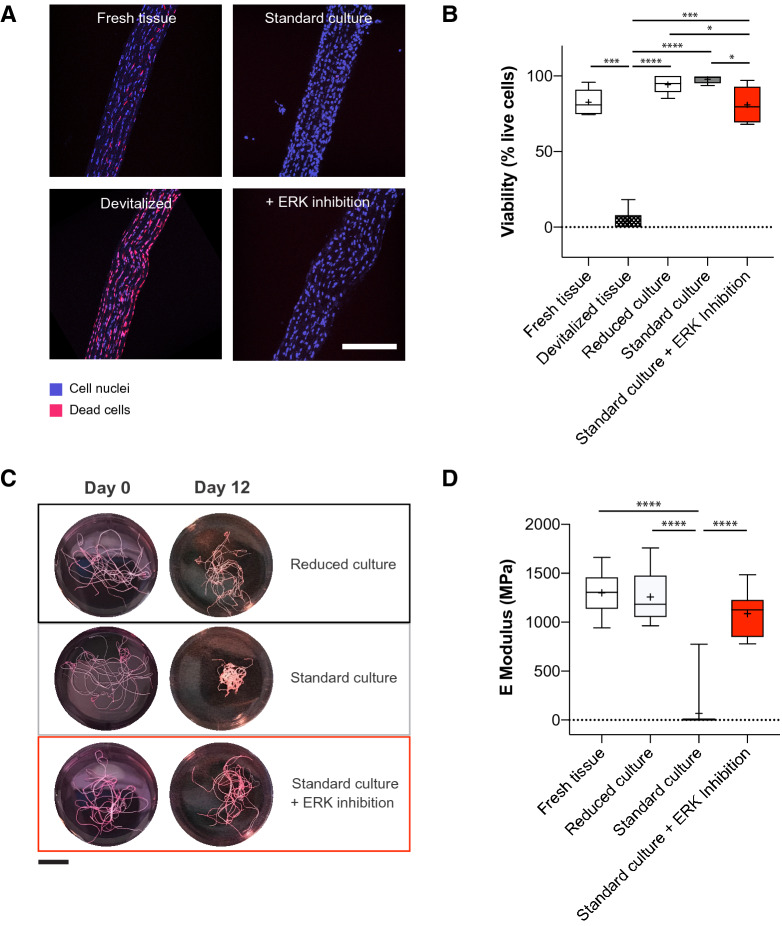
Inhibition of ERK 1/2 activity prevents loss of tendon biomechanical properties in tendon explants cultured for 12 days. (**A**) Representative fluorescence microscopy images of tendon fascicles with nuclear staining of all cells (blue) and dead cells (EthD-1, violet). Scale bar 200 μm. (**B**) Quantification of cell viability in uncultured tendon explants (fresh tissue), methanol treated uncultured negative control tendons (devitalized tissue) and ex vivo cultured explants in reduced and standard culture treated without and with an ERK 1/2 inhibitor, N = 6. Devitalized tissue significantly differs from all other conditions. (**C**) Representative macroscopic images of tendon fascicles before treatment (uncultured tendon explants, day 0) and after 12 days of incubation in indicated culture conditions. Scale bar 10 mm. (**D**) Elastic moduli of isolated tendon fascicles (freshly isolated or cultured ex vivo in reduced conditions, standard conditions or treated with an ERK 1/2 inhibitor), N = 12 (N = 10 for standard culture, due to tissue rupture during the mounting procedure. Negative values of elastic modulus resulting from preliminary tissue failure were set to zero). The standard culture significantly differs from all other conditions. GraphPad Prism (version 8.4.3) was used to perform statistical analyses and generate the figures. Box plots (25th and 75th percentiles) with whiskers (5th and 95th percentiles), median (line) and mean (+). Bar plots represent mean values + SD. All statistical tests unless otherwise stated: Repeated measures ANOVA with Tukey's multiple comparisons test with *p < 0.05, ***p < 0.001, ****p < 0.0001.

To further delineate the role of ERK 1/2, we pharmacologically inhibited their downstream activity using a small molecule inhibitor (SCH772984). First, we tested whether ERK inhibition affects viability in tendon explants (Fig. 2A,B). We observed a high cell viability (> 80%) in all conditions except for the methanol devitalized control tissue (Fig. 2A,B). These results show that the ERK inhibitor has no cytotoxic effects, and that the ex vivo culture retains the viability of the tendon fascicles. In contrast to fascicles cultured under standard conditions, ERK inhibition suppressed macroscopic contraction of fascicles after 12 days of culture similar to fascicles cultured under reduced, non-degrading conditions (Fig. 2C). Next, we assessed the functional properties of tendon explants (Fig. 2D). Mechanical testing revealed a previously observed, drastic drop in elastic moduli (EMod) of tissues maintained in standard culture (mean: 70 MPa) when compared to fresh tissue (mean: 1300 MPa) and reduced culture conditions (mean: 1260 MPa)^12^. Surprisingly, ERK inhibition fully rescues the mechanical properties in standard conditions (mean: 1090 MPa). Additionally, we tested whether mechanical unloading alone was responsible for ERK 1/2 phosphorylation and the loss of functional properties in standard conditions. Hence, we cultured tendon fascicles under static load conditions using a custom-made loading device. While static mechanical load fully prevents fascicles from losing their mechanical properties, ERK 1/2 were phosphorylated under this loaded ex vivo condition (Supplementary figure S1A, S1B).

**Figure 3:**
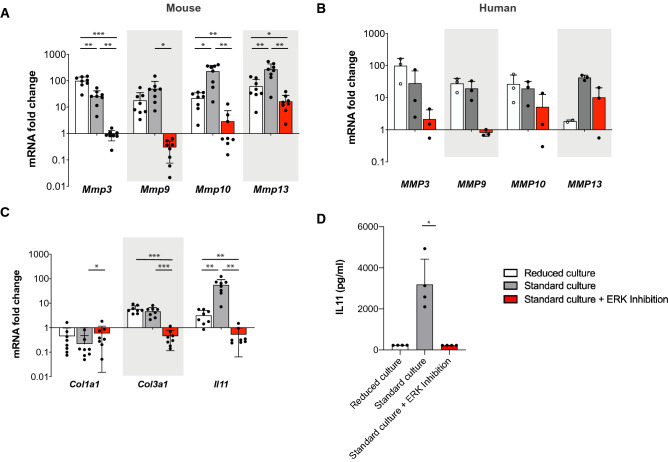
Inhibition of ERK 1/2 activity prevents the induction of matrix metalloproteinases, collagen type 3 and interleukin 11 gene expression in tendon explants. (**A**) Gene expression of murine *Mmps* after 12 days ex vivo culture. Data are normalized to 2 reference genes, N = 8. (**B**) Gene expression of human *MMPs* after 3-days ex vivo culture. Data are normalized to 2 reference genes, N = 3. (**C**) Gene expression of *Col1a1, Col3a1* and *Il11* in murine tissue after 12 days ex vivo culture. Data are normalized to 2 reference genes, N = 8. (**D**) Detection of IL11 protein levels in supernatants obtained from cultured tendon fascicles by ELISA, N = 4. Friedman test with Dunn’s multiple comparison test. Gene expression data is plotted on a logarithmic scale. GraphPad Prism (version 8.4.3) was used to perform statistical analyses and generate the figures. Box plots (25th and 75th percentiles) with whiskers (5th and 95th percentiles), median (line) and mean (+). Bar plots represent mean values + SD. All statistical tests unless otherwise stated: Repeated measures ANOVA with Tukey's multiple comparisons test with *p < 0.05, **p < 0.01, ***p < 0.001.

To examine how ERK inhibition suppresses tissue breakdown in tendon fascicles, we measured the gene expression of ECM degrading enzymes (matrix metalloproteinases *Mmp3*, *9*, *10*, *13*), which we had identified previously by RNA-sequencing and mass spectrometry-based proteomics^12^ (Fig. 3A). The expression of all measured MMPs was strongly increased under standard conditions compared to the fresh, uncultured control tissue (*Mmp3*: 26-fold, *Mmp9*: 50-fold, *Mmp10*: 230-fold, *Mmp13*: 269-fold). Non-degrading, reduced culture showed generally lower up-regulation of MMPs than standard culture. Strikingly, when ERK 1/2 activity was inhibited in standard culture conditions the expression of *Mmp3*, Mmp*9*, and Mmp*10* was not induced and fully remained at baseline level of fresh control tissue. For *Mmp13* we found that its expression was increased even in the ERK inhibition condition (16-fold) but was much lower than in the ex vivo standard condition (269-fold). To verify that these effects occur in other tendon types and in human tissue, we cultured human tendon tissues ex vivo and measured MMP expression. Indeed, we could confirm the MMP induction and inhibition in human tendons without and with ERK-inhibition, respectively (Fig. 3B). Collectively, these data show that ERK inhibition prevents the induction of ECM degrading enzymes (MMPs) and the loss of mechanical properties in tendon explants.

To investigate whether ERK influences further components of degenerative ECM remodeling we assessed the gene expression of collagen type 1 (*Col1a1*), collagen type 3 (*Col3a1*) and interleukin 11 (*Il11*) (Fig. 3C). Expression of collagen type 1, the most abundant component of the healthy tendon ECM, was reduced in all ex vivo conditions including when ERK 1/2 was inhibited. However, ERK 1/2 inhibition partially rescues the expression of *Col1a1*. In contrast, collagen type 3 expression, which is a known early phenotypic marker in tendinopathic ECM remodeling^19–21^, was increased in tendon fascicles. Interestingly, blocking ERK 1/2 inhibited the increased expression of *Col3a1*. We have previously observed that interleukin 11 (*Il11*), a stromal-derived cytokine and strong fibrosis driver in many tissues^22–24^, might be an important cytokine for tendon deterioration as it was heavily up-regulated in mechanically degraded tissues compared to non-degrading conditions (Supplementary figure S2A). Here, we show that *Il11* was increased in degrading fascicles (Fig. 3C), and required ERK 1/2 activity to be expressed and released into the supernatant (Fig. 3D). When further confirming that *Il11* is only expressed in degraded tendon tissue, we observed that mechanical loading of the tissue fully blocks the protein expression of IL11 (Supplementary figure S2B).

## Discussion

The molecular events leading to tendon degeneration are poorly understood. Mechanical unloading of tendon sub-structures due to tissue rupture or microdamage is thought to be an ignition switch of the tissue breakdown cascade^16,20,25,26^. Here, we show that the ERK pathway drives tendon matrix degradation in mechanically unloaded tissue in a culture niche-dependent manner. We found that ERK 1/2 are highly phosphorylated in degrading tendon fascicles, and that ERK 1/2 activity is required for tendon deterioration. However, ERK 1/2 phosphorylation alone is not sufficient to induce loss of tendon mechanics as mechanically loaded explants show ERK 1/2 phosphorylation but retain their mechanical properties. These findings nicely show that biomechanical and biochemical cues act together in the maintenance of tendon mechanics and functional properties. In addition, the strong increase of MMP gene expression under the degrading conditions of a vascular-like niche depends on ERK 1/2 activity. We have previously shown that under standard culture conditions reactive oxygen species (ROS) are implicated in the MMP-driven tendon breakdown in load-deprived explants^12^. Since ERK is known to mediate ROS stress^27^, it seems plausible that a ROS-ERK-MMP axis pathway is a central mechanism of tendon tissue breakdown. Our data, establishing a link between ERK, MMPs and tendon matrix degradation, are in line with previous work reporting the control of MMP expression through ERK 1/2 in cultured fibroblasts, smooth muscle cells and cancer cells^28–31^.

Our finding showing that ERK 1/2 inhibition protects tendon tissue from degradation is in agreement with previous studies reporting the involvement of ERK 1/2 in early tendinopathy. Using cultured human tenocytes, ERK 1/2 have been recognized to be key signal transducers between pathological triggers and tendinopathic phenotypes. For instance, Morita et al. showed that ERK 1/2 mediates the IL1β induced fibrotic phenotype of diseased tendon cells^32^. Furthermore, IL17A, IL33 and hypoxia were all shown to induce the expression of inflammatory cytokines and collagen type 3 in human tenocytes in an ERK-dependent manner^19,21,33^. Accordingly, we found that ERK 1/2 inhibition abrogates collagen type 3 expression, which is induced in load-deprived tendon explants. Similarly, we found that the stromal cytokine IL11, which is a potent fibrosis driver and therapeutic target in many tissues^22–24^, is highly upregulated in degrading tendon tissue but not when ERK 1/2 was blocked. This finding mirrors previous reports by Nishina et al*.* which demonstrated that the ERK pathway mediates ROS-induced production of IL11 during early events of acute damage in hepatic stroma^34^.

In this study we focused on gene expression, functional read-outs and ERK 1/2 protein levels using the fascicle ex vivo explant system as it allows for the controlled parametric investigation of the load-bearing tendon stromal compartment. Although the relatively simple structure of tail tendon fascicles offers advantages for the study of isolated cellular mechanisms, we acknowledge that there are limitations with using this model. In tendon fascicles the ratio of ECM to cells is very high and measuring ECM turnover, in particular cellular collagen, at the protein level by bulk methods is difficult due to these high ECM baseline amounts. Although we cannot rule out that the observed decrease in collagen type I gene expression in standard culture is responsible for the measured loss of tissue stiffness, we know from our previous proteomic quantification studies^12^ that the overall content of collagen type 1 protein does not significantly differ between standard and reduced culture despite clear differences in ERK 1/2 phosphorylation state. Further, recent literature has revealed that tendon is a complex tissue with heterogeneous cell populations, including various stromal, endothelial and immune cells^35,36^. Crosstalk between the cells in tendon core and the surrounding synovial-like sheath is vital for tendon physiology and healing mechanisms^20,37^. The finite synovial tissue layer is often damaged during fascicle extraction ex vivo which compromises the cross-compartmental signaling^12,37^. Furthermore, murine tendons differ from their human counterparts in terms of function, composition and cross-linking patterns, with the mechanical and structural properties of tail tendons significantly differing from those of energy-storing tendons^38,39^. Nonetheless, there are compelling similarities between the murine tail tendon and human load bearing tendon, both showing an ERK-dependent MMP regulation that strongly resonates with previous studies on diseased human tendons. In this sense, the present work underscores the utility of the ex vivo fascicle model for dissecting the early molecular events that may precede the clinical presentation of chronic tendon pathologies. Future ongoing work is envisioned to investigate the role of ERK 1/2 pathway in degenerated human tendons using patient-derived materials from different anatomical locations.

## Experimental procedures

### Kinase enrichment analysis

Upstream protein kinases associated with the DEG from dataset (ArrayExpress: E-MTAB-7832)^12^ were identified using Expression2Kinases suite (MacOS, Version 1.6.1207, http://www.maayanlab.net/X2K/)^40^. Briefly, the top 3000 DEG were fed to Expression2Kinases pipeline which applies enrichment analysis to infer and rank potential transcription factors regulating the quired genes. Next, it identifies binding partners and constructs protein–protein transcriptional regulatory subnetwork, which are loaded to the Kinase Enrichment Analysis (KEA) module^41^. The identified kinases were mapped to kinome tree dendrogram using Coral tool and phylogenetic information derived from Manning et al*.*^42,43^ (http://phanstiel-lab.med.unc.edu/CORAL/. Source code is available at https://github.com/dphansti/CORAL).

### Murine tissue explantation and load-deprived culture

Twelve 11-weeks-old wild-type C57BL/6 male and female mice were obtained from the ETH Phenomics Center (EPIC) and euthanized with CO_2_ previous to tissue harvest. Tendon fascicles were cautiously extracted from the murine tails using a surgical clamp. For fresh tissue controls we used untreated fascicles directly after extraction. For ex vivo culture and treatments, fascicles were immediately hydrated in PBS after extraction and randomly distributed among the test groups. Subsequently, fascicles were maintained deprived from load in 6-well plates filled with 2 ml culture medium for 12 days in a humidified atmosphere at 5 kPa/5% CO_2_ and at 37 °C, 20 kPa/20% O_2_ (standard culture) or at 29 °C, 3 kPa/3% O_2_ (reduced culture). As culture medium we used high glucose Dulbecco's Modified Eagle's Medium (Sigma Aldrich, D6429) containing 10% FBS (Gibco #10500-64 heat inactivated), 1% Penicillin–Streptomycin (Sigma Aldrich, P0781) and 200 µM ascorbic acid (Wako Chemicals 013-19641). Medium was additionally supplemented with an ERK 1/2 inhibitor (5 µM, SCH772984, Selleck S7101) from the beginning of the experiment. SCH772984 blocks the ERK auto activation loop and the phosphorylation of the downstream target ribosomal S6 kinase. Fascicles for mechanical testing and viability were cultured individually, while fascicles for gene expression and protein analysis were pooled (up to 15 fascicles with a maximum length per fascicle of approximately 50 mm). Medium was changed after 6 days. All experimental readouts were performed after 12 days of ex vivo culture. The Ethics Committee of the Cantonal Veterinary office of Zurich ethically approved all animal experiments under the permit number ZH239/17. All experiments were performed in accordance with relevant local guidelines and regulations and in compliance with the ARRIVE guidelines^44^.

### Tissue culture under mechanical load

Fascicles from 5 different mice were clamped at a length of 20 mm and their mechanical properties were measured before (day 0, fresh tissue) and after (day 12, static loading) cultivation under static mechanical load using a custom-designed uniaxial stretcher (see section “Biomechanical testing”). Fresh, clamped fascicles were placed into silicon chambers (filled with 2 ml medium) and cultured in a custom-designed bioreactor^45^ at crimp disappearance length (static load) and under standard culture conditions. Crimp disappearance length was visually set for each fascicle using a stereomicroscope (Nikon SMZ 745T). As control, load-deprived fascicles (n = 5) were cultured in standard conditions as described above and mechanical tests were performed after 12 days of culture.

### Human tissue explantation and culture

Hamstring tendon pieces (Gracilis, Semitendinosus) were collected from male and female patients undergoing anterior cruciate ligament reconstruction surgeries (mean age: 28 years, sd: 10.7). Immediately after dissection, tendons were immersed in standard culture medium and cut into smaller pieces (3–5 mm × 5–10 mm). Small pieces were either snap frozen as fresh control or subsequently cultivated in a 6-well plate with 5 ml culture medium for 3 days in standard culture or reduced culture (see section “Murine tissue explantation and load-deprived culture”). As culture medium we used Dulbecco's Modified Eagle's Medium/Nutrient Mixture F12 (Sigma Aldrich, D8437) containing 10% FBS (Gibco #10500-64 heat inactivated), 1% Penicillin–Streptomycin (Sigma Aldrich, P0781) and 200 µM ascorbic acid (Wako Chemicals 013-19641). Medium was additionally supplemented with an ERK 1/2 inhibitor (5 µM, SCH772984, Selleck S7101) from the beginning of the experiment. The Cantonal Ethics Commitee of Zurich reviewed and ethically approved all experiments under the permit number 2020-01119. Experiments were conducted according to the approved guidelines and tissues were collected with signed patient consent.

### Biomechanical testing

Mechanical testing was performed according to previously described methods^12^. Briefly, force–displacement data were recorded from 12 individually clamped fascicles (cut to a length of 20 mm after cultivation) from different mice on a custom-designed uniaxial stretcher. Fascicles were kept hydrated between Kapton films (DuPont, 25 µm thickness) during mechanical testing. Average diameter was determined previous to treatment to calculate the cross-sectional area. Nominal stress was calculated based on this initial cross-sectional area and the pre-load length at 0.015 N corresponded to 0% strain. Tangential elastic modulus was calculated after 5 preconditioning cycles in the linear part of the stress–strain curve (0.5–1% strain) by fitting a linear slope (Matlab R2016a, Version 9.0.0.341360). Any spurious negative values of elastic modulus resulting from severe tissue failure during mechanical characterization were set to zero to accord with the total functional impairment of the tissue.

### Cell viability analysis

To measure cell viability, dead cells were stained with Ethidium Homodimer-1 (EthD-1) solution (2 mM, AS 83208, Anaspec) in PBS for 10 min. Cells in negative controls were devitalized by treatment with 100% methanol (MeOH) for 10 min. Fascicles were thoroughly washed in PBS after EthD-1 and MeOH treatment and subsequently fixed in 10% formalin for 20 min, previous to nuclear staining with NucBlue reagent (1 drop/ml, R37606, Thermo Fisher). Stained fascicles were embedded in a 50% Glycerol:PBS solution and Z-stacks of 40 µm height (9 stacks by 5 µm) were acquired on a laser scanning confocal microscope (A1R Nikon) in triplicates per fascicle by using a 20 × objective (Nikon Ti2) (https://www.microscope.healthcare.nikon.com/en_EU/products/software/nis-elements). Quantification of the viability was performed using ImageJ (Fiji version 2.0.0-rc-59/1.51n, https://imagej.net/). We separated the channels and maximum projected all images of a stack into a single image before subtracting background using the *rolling ball* method with a ball radius of 20 pixels. Next, we adjusted image contrast/brightness and converted the image into 8-bit grayscale to then binarize it. To avoid counting cell clusters instead of single cells, we watershed the image. Finally, we used the *analyze particles* tool to count the imaged dead cells (stained with EthD-1, size: 10–200 pxn^2^) and all imaged cells (stained with NucBlue, size: 30–200 pxn^2^). Percentage cell viability was calculated by dividing the number of live cells (nuclei stained with NucBlue only) by the total number of cells (nuclei stained with NucBlue only plus nuclei stained with EthD and NucBlue).

### RNA extraction and quantitative real-time PCR

Fascicles from three mice were pooled for each N of gene expression analysis. RNA was extracted from 10–15 murine fascicles and 70–90 mg of human tissue adapting a formerly established protocol^12^. In summary, tissues were snap frozen in liquid nitrogen, ground in 100 µl GENEzol (Labforce LSBio, 4402-GZR200) in a cryogenic mill (Spex6775 FreezerMill) for 2–4 milling cycles at 15 cps and lysed in 1–1.5 ml GENEzol. To completely homogenize the tissue and remove insoluble ECM components, human samples were passed ten times through a 21G syringe needle and were centrifuged at 5000 *g* for 5 min. Next, chloroform was added at a ratio of 1:5 from the extract volume and the lysate was centrifuged (12,000 *g*, 15 min, 4 °C). The RNA containing upper (aqueous) phase was obtained, mixed with the equal amount of 70% EtOH and loaded onto the PureLink silica membrane column. Total RNA was isolated using the PureLink RNA Micro Scale Kit for murine tissue (Invitrogen, 12183016) and the PureLink RNA Mini Scale Kit for human tissue (Invitrogen, 12183018) according to the manufacturer’s instructions. 100–400 ng of RNA was transcribed into 40 µl cDNA using the High-Capacity cDNA Reverse Transcription Kit (Applied Biosystems, 4368814). qRT-PCR was performed using 1.5 µl cDNA and the KAPA PROBE FAST Master Mix (Sigma-Aldrich, KK4707). Gene expression was carried out using the StepOnePlus Real‐Time PCR System (Applied Biosystems) and the following TaqMan probe/primer sets: *Col1a1* (Mm00801666_g1), *Col3a1* (Mm01254476_m1), *Mmp3* (Mm00440295_m1), *MMP3*, (Hs00968305), *Mmp9* (Mm00442991_m1), *MMP9* Hs00957562), *Mmp10* (Mm01168399_m1), *MMP10* (Hs01055413), *Mmp13* (Mm00439491_m1), *MMP13* (Hs00942584) and *Il11* (Mm00434162_m1). Relative gene expression was calculated by the comparative Ct method by normalizing the data to the reference genes *Anxa5* (Mm01293059_m1), *ANXA5* (Hs00996186) and *Eif4a*2 (Mm01730183_gH), EIF4A2 (Hs00756996) or RPL13A (Hs04194366) and to freshly isolated fascicles.

### Interleukin 11 ELISA

The supernatants (2 ml) from 10–15 fascicles pooled from 3 mice were collected and stored at − 80 °C for further use. Samples were 4 × diluted in sample buffer and interleukin 11 was detected by a SimpleStep ELISA following the manufacturer’s instructions (Abcam, ab215084).

### Western blot analysis

Fascicles from three mice were pooled for each N of protein analysis. 10–15 fascicles were thoroughly washed in PBS and snap frozen in liquid nitrogen previous to lysis in RIPA buffer (Sigma-Aldrich, R0278; supplemented with a phosphatase inhibitor, Thermo Fischer, A32957). Fascicles were homogenized with surgical scissors in 50 µl ice-cold RIPA and lysed for 10 min on ice with 3 times vortexing the samples for 10 s. Subsequently, lysates were centrifuged (20,000 *g*, 10 min, 4 °C) and equal protein amounts (8 μg) were boiled in 6 × Laemmli sample buffer including 2-mercaptoethanol (10 min, 95 °C, Alfa Aesar, J61337). Next, Mini-PROTEAN TGX stain-free protein gels (4–15%, Bio-Rad, 4568086) were used to separate proteins before they were transferred on to polyvinylidene fluoride (PVDF) membranes using the Trans-Blot Turbo System (Bio-Rad). Membranes were blocked in nonfat dry milk/TBS-T (5%, 30 min, RT) and primary antibodies were incubated in BSA/TBST-T (5%) overnight at 4 °C. After washing the membranes three times in TBS-T they were incubated with the corresponding horseradish peroxidase (HRP)-conjugated secondary antibodies in nonfat dry milk/TBS-T (2.5%, 60 min, RT). The UltraScence Pico Ultra Western Substrate (GeneDireX, CCH345-B) and the ChemiDoc MP imaging system (Bio-Rad) were used to visualize HRP.

Antibodies used in this study were: rabbit anti-ERK 1/2 (1:1000, Cell Signaling 9102), rabbit anti-phospho-ERK 1/2 (1:1000, Cell Signaling 9101), mouse anti-α-tubulin (B-5-1-2, 1:3500, Santa Cruz Biotechnology sc 23948), goat-anti-mouse-HRP (1:15 000, Sigma-Aldrich, SAB3701073-2), and goat-anti-rabbit-HRP (1:15 000 Sigma- Aldrich, SAB3700878-1). For each experiment, the membrane was first probed for phospho-ERK 1/2, then stripped by a Western blot stripping buffer (Restore Plus, Thermo Fisher 46,430) and finally probed for total ERK 1/2. Afterwards, the membrane was developed for α-tubulin. For quantification, signal intensities were measured using the *gels tool* of ImageJ (Fiji version 2.0.0-rc-59/1.51n, https://imagej.net/) and phosphorylation levels were calculated by normalizing the values of phospho-ERK 1/2 to total ERK 1/2.

### Statistical analysis

Statistical tests were performed using GraphPad Prism (version 8.4.3, https://www.graphpad.com/scientific-software/prism/). Between group differences of normally distributed datapoints were evaluated by repeated measure ANOVA tests with the Tukey’s post hoc multiple comparison method. Differences in IL11 secretion between culture conditions were evaluated using the non-parametric Friedman test and Dunn’s post hoc multiple comparison test. In all cases, significance was defined as *p < 0.05, **p < 0.01, ***p < 0.001, ****p < 0.0001. Data were represented either as box and whisker plots with individual data points, showing 25th and 75th percentiles (box) and 5th and 95th percentiles (whiskers) or bar charts (showing mean + SD). Negative values of viability and elastic moduli resulting from limitations in the quantification analysis were set to 0.

## Supplementary Information

Supplementary Information 1.

Supplementary Information 2.
