# Supplementary material for: Inhibition of ERK 1/2 kinases prevents tendon matrix breakdown
Source: Sci Rep. 2021 Mar 25;11:6838. doi: 10.1038/s41598-021-85331-1 (PMC7994809; doi:10.1038/s41598-021-85331-1)
Supplement: Supplementary file 2 — Supplementary Information 2. [file 41598_2021_85331_MOESM2_ESM.docx]

Supplementary information

**Title:** Inhibition of ERK 1/2 kinases prevents tendon matrix breakdown

**Autors:** Ulrich Blache^1,2 #^, Stefania L. Wunderli^1,2 #^, Amro A. Hussien^1,2^, Tino Stauber^1,2^, Gabriel Flückiger^1,2^, Maja Bollhalder^1,2^, Barbara Niederöst^1,2^, Sandro F. Fucentese^1^ and Jess G. Snedeker^1,2 *^

**Affiliation:**

^1^ Balgrist University Hospital, University of Zurich, Zurich, Switzerland

^2^ Institute for Biomechanics, ETH Zurich, Zurich, Switzerland

^#^ UB and SLW contributed equally to this work

**Supplementary Tables**

Supplementary table 1 (“ST1_Kinase_Enrichment_Analysis.xlsx”)

**Supplementary Figures**

**Figure 1**

**
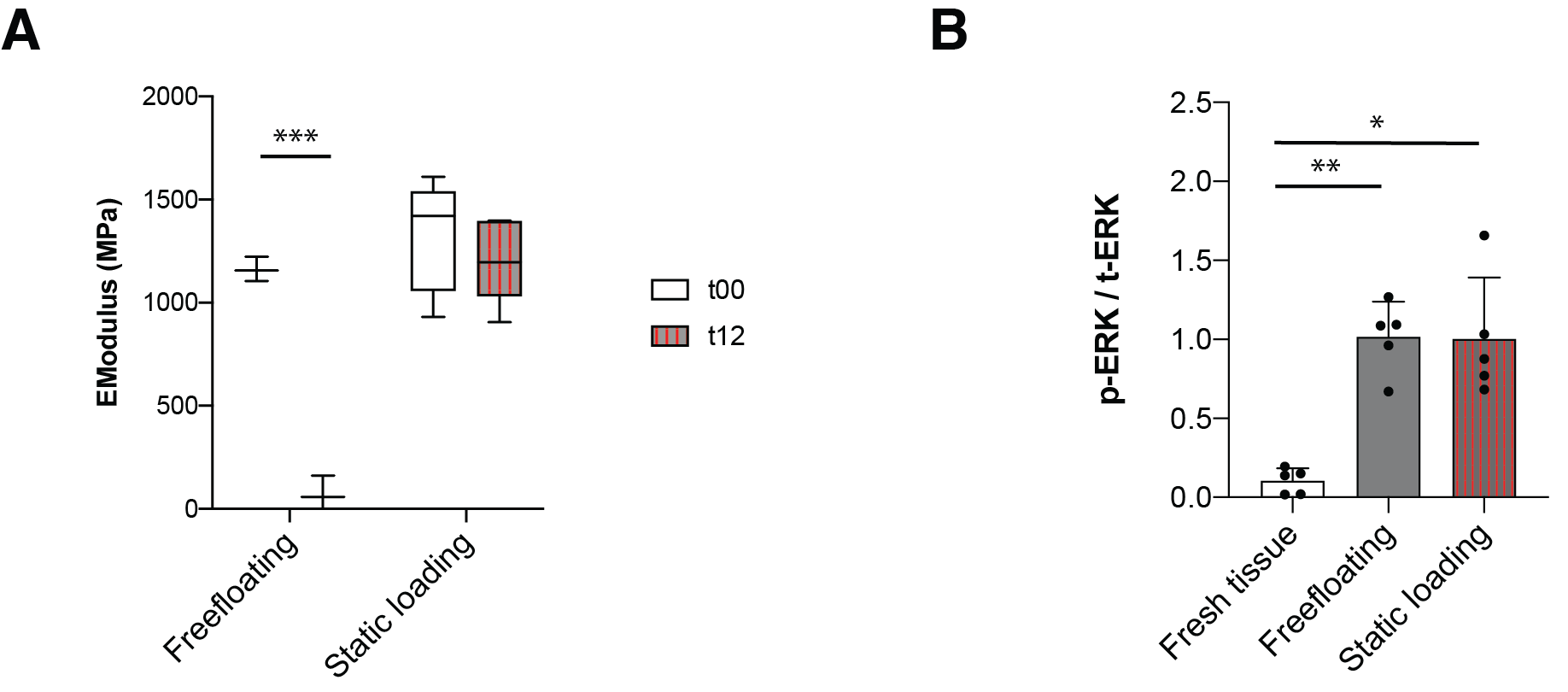
**

**Supplementary figure S1: Effect of mechanical loading on tissue mechanics and ERK phosphorylation in fascicles cultivated under standard culture conditions** (A) Elastic moduli of isolated tendon fascicles (freshly isolated or 12-days *ex vivo* cultured free from load or under static load at crimp disappearance), N=5 (N=3 for freefloating culture, due to tissue rupture during mounting procedure). Negative values of elastic modulus resulting from preliminary failure were set to zero). Freefloating culture significantly differs from fresh tissue control and static loading condition. Box plots (25th and 75th percentiles) with whiskers (5th and 95th percentiles) and median (line). Statistical tests: Mixed-effects analysis of two-way ANOVA with repeated measures with *** p<0.001. (B) Quantification of Western blot band pixel intensities of phosphorylated ERK (p-ERK 1/2, ERK 1: Thr202/Tyr204; ERK 2: Thr185/Tyr187) compared to the total ERK (t-ERK) in freshly isolated or cultured tendon fascicles (12 days). GraphPad Prism (version 8.4.3) was used to perform statistical analyses and generate the figures. Bar plots represent mean values + SD. Statistical tests: Repeated measures ANOVA with Tukey's multiple comparisons test with * *p* < 0.05, ** p<0.01.

**Figure 2**

**
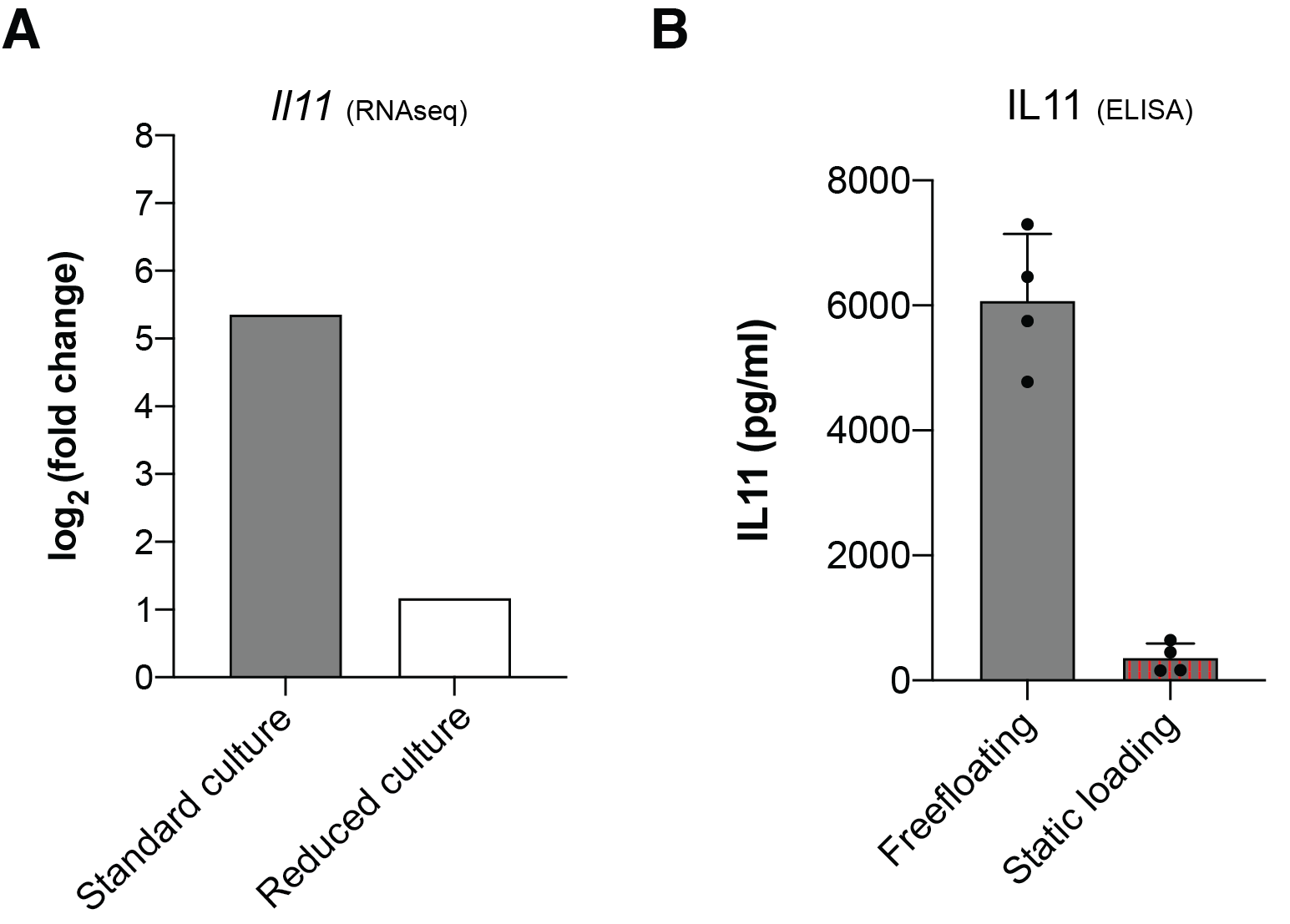
**

**Supplementary figure S2: IL11 is induced in conditions that show tissue degradation** (A) *Il11* gene expression in degrading conditions (standard culture) when compared to fresh control tissue (log2(fold change) = 0), but not in mechanically intact fascicles (reduced culture) (data is reproduced from the RNAseq dataset in Wunderli et al. 2020, Matrix Biology ^12^), n=3. (B) IL11 in supernatants of degrading (standard culture freefloating) and non-degrading (standard culture statically loaded) fascicles measured by ELISA, n= 4. GraphPad Prism (version 8.4.3) was used to generate the figures.

**Figure 3**

**Supplementary figure S3: Full Blot Images of Western Blots.** Monochrome inversion of original Western blot images for tubulin (A), phospho ERK (B) and total ERK (C) used in figure 1D of the main manuscript. Squares with dashed lines delineate the cropped regions of the images used in the main figure. Exposure times for tubulin, phospho ERK and total ERK were 23.6s, 34.7s, 37.9s, respectively.
